# Reducing opioid related deaths for individuals who are at high risk of death from overdose: a co-production study with people housed within prison and hostel accommodation during Covid-19

**DOI:** 10.3389/fpubh.2023.1080629

**Published:** 2023-06-15

**Authors:** Anne Campbell, Sharon Millen, Li Guo, Uisce Jordan, Amanda Taylor-Beswick, Chris Rintoul, Aisling Diamond

**Affiliations:** ^1^SSESW, Queens University Belfast, Belfast, United Kingdom; ^2^Manchester Metropolitan University, Manchester, United Kingdom; ^3^Faculty of Health, Social Care and Medicine, Edge Hill University, Ormskirk, United Kingdom; ^4^University of Cumbria, Lancaster, United Kingdom; ^5^Cranstoun, St. Andrew's House, Surbiton, United Kingdom; ^6^Southern Health and Social Care Trust, Craigavon, United Kingdom

**Keywords:** Opioid, overdose, wearable device, co-production, injecting drug use

## Abstract

**Background:**

A record number of Opioid-related deaths occurred in Northern Ireland in 2021 and it is acknowledged that the Covid-19 pandemic compounded drugs related deaths crisis. This co-production study set out to refine the design of a wearable device for Opioid users to detect and subsequently prevent a potential overdose situation.

**Method:**

Purposive sampling was used to recruit people who had substance use disorders and were living in a hostel and prison during the Covid-19 pandemic. Principles of co-production influenced the study, which encompassed a focus group phase and a wearable phase. The initial phase included three focus groups with participants who inject Opioids and one focus group with workers from a street injector support service. During the wearable phase, the participant group tested the feasibility of the wearable technology in a controlled environment. This included testing the transferability of data from the device to a backend server on the cloud.

**Results:**

All focus group participants expressed an interest in the wearable technology when it was presented to them and agreed, that in principle, such a device would be extremely beneficial to help reduce the risk of overdose within the active drug using community. Participants outlined factors which would help or hinder the design of this proposed device and their decision to wear it, if it were readily available to them. Findings from wearable phase indicated that it was feasible to use a wearable device for monitoring Opioid users’ biomarkers remotely. The provision of information regarding the specific functionality of the device was considered key and could be disseminated via front line services. The data acquisition and transfer process would not be a barrier for future research.

**Conclusion:**

Understanding the benefit and disadvantages of technologies such as a wearable device to prevent Opioid-related deaths will be critical for mitigating the risk of overdose for people who use Heroin. It was also clear that this would be particularly relevant during Covid-19 lock-down periods, when the effects of the pandemic further exacerbated the isolation and solitude experienced by people who use Heroin.

## Background

New figures from the Northern Ireland Statistics and Research Agency (NISRA) show that 213 people died because of a drug related deaths in 2021 (1). Drug-related deaths have increased from 102 deaths in 2011 to 218 in 2020 and 213 deaths in 2021.

Deaths involving Heroin, Morphine and Cocaine were the highest on record in 2020 in Northern Ireland and drug-related deaths have more than doubled in a decade (1). This mirrors a nationwide rise in drug-related deaths, which can be attributed to macro-environmental changes that began during the Covid-19 pandemic, such as increased physical isolation, the increased rate of solitary Opioid use, mental health stressors, economic uncertainty, and enhanced lethality of the drug supply (2–4). Whilst there has not been a noticeable increase in Fentanyl or Nitazines, it is clear that an increase in polydrug use including “street” and research benzodiazepines has been noted in a recent report from the Northern Ireland Alcohol and Drug Alliance (5).

The Covid-19 pandemic disproportionally impacted People Who Inject Drugs (PWID) because they are a population subject to pre-existing socio-structural inequalities such as economic disadvantage, stigma and housing inequalities (6–8). Within the population of PWID, users who are homeless and those who are recently released from prison are considered particularly at risk of an Opioid overdose (9). Information from the Street Injectors Project, Extern (SISS), who work with PWID in Northern Ireland, indicates that there has been a steady increase in the reported intravenous (IV) use of Heroin alongside an increase in the IV use of Cocaine. There has also been more emphasis on Heroin mixed with street manufactured benzodiazepines. Wakeman et al. (4) noted that people with Opioid use dependency may have sought other substances during the Covid-19 pandemic, such as alcohol and benzodiazepines, both of which potentiate overdose risk. To date, there has been limited research in this area but figures suggest that a suite of interventions is required to help reduce the number of fatalities amongst an ever-expanding Opioid problem (10).

The majority of premature deaths of PWID are preventable through Opioid substitution programs and using Opioid reversal drugs such as Naloxone (11). It is imperative that innovative interventions are sought, tested, and implemented to address the complex issue of drug-related deaths (12) and the use of technology has been identified as a means of supporting and replacing traditional interventions in various contexts (13, 14). Wearable technology has great potential in the Harm Reduction space to detect overdose, particularly when people use alone, but has been underutilized (15). A growing body of literature demonstrates the potential of digital interventions, including wearable devices, for preventing opiate overdose (2, 16–19).

Digital technologies could also assist with advancing theories of human behavior by generating opportunities to test mediators of change and identify the most potent intervention components (20, 21). Accordingly, researchers have sought new theoretical approaches to guide the use of digital technologies that enable intensive longitudinal data collection, tailoring to participants’ specific needs, or delivering targeted feedback or prompts in response to changing conditions (22, 23). Well-designed and rigorously tested digital interventions, grounded in relevant behavior theories, hold exciting promise for supporting the mental and physical health needs of individuals living with opiate dependency.

In order to address existing inequalities, consultations with PWID advocacy groups are vital when designing inclusive health response to the Covid-19 pandemic (24). The co-production study described in this article focuses on working with PWID to adapt wearable technologies to prevent loss of life in highly vulnerable populations. In this context, we worked co-productively with Opioid users from relevant frontline services to obtain their views and ideas regarding the design of the technology and also to test the feasibility of wearing such a device. Whilst working co-productively with the participant group, we aimed to refine the design of a wearables device that would specifically address these current concerns by monitoring Opioid users’ life sign (SPo2 and heart rate) in order to ascertain if these could be sent successfully to a back-end server housed at Manchester Metropolitan University.

## Method

### Study overview and sample

We used a two-phase approach to answer two research questions. To examine the first question, “*what are the views and ideas of a prison population and homeless hostel occupants with Opioid disorders regarding the feasibility and acceptability of the wearable device?*” We conducted three focus groups. For the second question, “*can the data from the wearables device be successfully transferred to a secure server and what is needed (if anything) to improve the mobile technology*?,” we conducted a “Wearables Phase.”

Both phases were guided by principles of co-production, a method of working where service providers and users collaborate to achieve a common goal. Working in this way helps refute the idea that people with lived experiences cannot take part on an equal footing with those who hold professional positions. The approach “is value-driven and built on the principle that those who are affected by a service are best placed to help design it” [(25), unpaginated]. Reflective of the co-production nature of the study we also conducted a small focus group (4 participants) remotely (via Zoom) with workers from a Street Injector Support Service (to obtain their perspectives on wearable technology in preventing Opioid overdoses).

The initial phase of the study encompassed the use of three focus groups in total with people who inject Opioids. Two were conducted face-to-face in a prison setting with people in prison; one in Hydebank College and one in HMP Maghaberry, whilst another was carried out face-to-face with service users who reside in a homeless hostel in Belfast. The number of participants ranged from 2 to 7 for each focus group. Two experienced researchers undertook the focus groups. One group was not recorded due to the researchers not being granted clearance from prison security to use a voice recorder. On this occasion, detailed notes were taken by the researchers.

In the second stage, we assessed the feasibility of wearing the device within a sample of the homeless population. Individuals were asked to wear the device whilst in the homeless shelter (for participant protection), whilst under the supervision of the researchers. Data was collected for 6 evening sessions between 10.11.21 and 29.11.21. Participants were asked to wear the device in the communal area between 6 and 8 p.m. during which time readings were taken from the device and stored on the individual wearable.

### Recruitment

Recruitment of the study sample of people in prison was coordinated by Alcohol and Drugs: Empowering People Through Therapy (ADEPT), a community sector organization that has been contracted to work with people who have identified drug use issues in prison. The purposive sample of hostel residents was recruited via the assistance of Extern (a non-governmental organization) who have management responsibility for the workers employed in the unit situated in Belfast. Sixteen (9 female) participants who had Opioid disorders were recruited to take part in the focus groups; 12 from the prison population and four from the homeless hostel. Their ages ranged between 22 and 43 years with a mean age of 29 years. In the wearable phase, there were six participants in the hostel (2 female) with an age range of 24–53 years.

Four members of staff at Extern’s Street Injector Service (SISS) were recruited through a gatekeeper at the organization. The focus group was conducted remotely (via Zoom) by an experienced researcher on the team.

### Informed consent

Issues of capacity and consent were routinely considered by the researchers involved and so they were able to identify any concerns about impaired mental capacity. Informed consent was sought at the beginning of each focus group by the researchers and each participant was asked to provide written consent after the Participant Information Sheet was read aloud to the group and all questions relating to the study were answered by the research team.

Prior to wearing the wristband for the designated 1.5–2-h period, staff checked the participant was still willing to wear the band and that informed consent was not marred by lack of capacity. The device was not used to prevent loss of life in this study. It was simply to gauge the service users’ views of the practicality of wearing the device and to assess the reliability of the transfer of data to a secure backend cloud service.

### Analyses

Focus groups were transcribed verbatim and anonymised through the removal of potential identifiers. The transcripts were uploaded to NVivo 12 for thematic analysis. All personal identifiers were removed from focus group transcripts prior to analysis to ensure that participants could not be identified, and the audio files immediately destroyed. Two members of the research team coded the qualitative data. From the codes, a series of broader themes was identified, and these form the overview of the qualitative findings.

Data from the wearables was transferred directly to a secure private server in the cloud and was monitored intermittently by a member of the research team. The transferrable data containing the two output readings (SPo2 and heart rate) did not contain any personal identifiers. After the completion of data collection, the information was downloaded from the server and analysed using Python.

## Findings 1: focus groups

### Focus groups with opioid injectors

The initial reaction from focus groups was positive, all participants expressed an interest in the wearable technology when it was presented to them. All participants agreed, that in principle, such a device would be extremely beneficial to help reduce the risk of overdose within the active drug using community. Almost three-quarters of those users taking part (11 out of 16) had at least one personal experience of overdosing whilst injecting Heroin and had been administered Naloxone on at least one occasion. Participants were asked what factors helped or hindered the design of this proposed device and their decision to wear it, if it was readily available to them.

#### GPS tracking

GPS tracking was the most common concern and biggest fear noted by the service users and all respondents (*n* = 16) were concerned about the GPS system within the device and its potential to track their everyday movements. All were keen to seek clarification regarding the particulars of the GPS system, for example, when this would be activated on the device, how, for what duration and exactly what information was captured by the technology?:

“I just wouldn’t like the tracker, I don’t know…I think that’s dangerous… would it be tracking you all the time or just when you have an overdose?… we’re always gettin’ into trouble, up to all f**king sorts on a daily basis, you wouldn’t want anyone to know what you’re doin, or where ya are like…” (Service User 3, female).

Three (out of 16) voiced significant concerns about wearing the device when using drugs at specific locations, for example, at their dealer’s house. Participants feared the GPS might be triggered when taking a ‘hit’ at such a location as this would attract unwanted attention to the dealer’s location. In such circumstances, they explained they would be likely to remove the device before injecting:

“*What if I’m lying up in my drug dealer’s flat and I’ve took a hit, and the thing goes off and then all these ambulances arrive, do you know what I mean …Like the ambulances will report that ‘til the police and people will think I’m a rat for getting the peelers* [police service]*…there’s people that have been left to die for less…”* (Service User 1, female).

The facilitators explained the GPS tracking would only be active if an individual’s blood Oxygen saturation levels dropped below 90 percent. A substantial decrease in the SPo2 level, as well as a decreased heart rate is considered abnormal and requires immediate medical attention. The participants were assured future versions of the wearable would utilize the life sign indicators on the wristband to trigger the activation of the GPS. The device would then be able to identify the location of the user at that particular time point only and share this location with Northern Ireland Ambulance Service (NIAS) so they could issue a rapid response vehicle. This significant and understandable fear of the GPS locator highlighted that the provision of accurate information regarding this function in particular was paramount in users wearing the device.

A significant number of participants (14 out of 16) raised concerns that linkage to the Northern Ireland Ambulance Service (NIAS) and response would attract unwanted attention from the police. They were unsure if they would want to take the risk of attracting attention from authorities for fear of the consequences such as being arrested or a return to prison if out on license. On the other hand, the linkage to NIAS was perceived to be essential by the majority, as it would ensure medical assistance would arrive in the quickest possible time:

*“I think it would be useful to link it up to the ambulance service as you’ve like only got so much time before ya pass [die] but not if it was linked up to the police….”* (Service User 6).

#### Wristband size

Over half of respondents (9 out of 16) noted the wrist size of active Opioid users is usually significantly smaller than the average person due to poor diet and sustained drug use. This was noted primarily by female respondents. Therefore, it was agreed by the participants that the wristband of the device should be designed to ensure the best fit possible:

*“Like most of us, by the time we get really bad…our wrists are smaller, like when I’m strung out, I’m only 6 or 7 stone like, you should make the strap out of elastic or whatever…”* (Service User 5, female).

One suggestion was to have a “snap band” as a strap or alternatively make it more flexible by using elastic. One respondent however, warned not to make the strap of the device a rubber band as users might be inclined to use this as a tourniquet when injecting. All participants agreed the device should not have a resale value.

#### Alarm

Participants also agreed the device should have an alarm which was activated in an overdose situation, to alert peers and/or passers-by to administer Naloxone which would be carried by the user. The alarm would consist of a loud tone as well as a voice message to administer Naloxone:

*“Like, what if you were down an alley somewhere havin’ a hit, I’ll give an example, if someone was down at the* [location] *in Belfast and there’s no-one around, but if someone was to walk past and that thing had an alarm, it could either be a really loud sound or a voice stating that, ‘I have Naloxone on my person, please administer the Naloxone, I am an Opioid user…’ if you’re on your own and you’ve overdosed, somebody needs to find ye …”* (Service User 7, female).

One respondent, who had personally experienced a number of Opioid overdoses suggested the device should also provide the user with a warning alarm when it detects early signs of potential overdose. Almost all (14 out of 16) felt an alarm on the device would be extremely useful to alert them if one of their peers was experiencing an overdose:

*“I think it would be really good if say you were with one of your friends and they went over and it was able to alert you as well and you had Naloxone and they didn’t…* (Service User 10, male).

#### “Cancel” button on device

One respondent spoke of how during his own experiences of injecting Heroin, he recovered from a near overdose situation several times and did not require any intervention. Thus, he suggested a button on the device to cancel an activation of a response from NIAS would be beneficial, not only for users but for the already overburdened ambulance service:

*“Do ya know the way some people are that close to overdose, but they come back? Like would there be a lot of false alarms? By the time the ambulance receives the alert and arrives and you’re alright and then you could’ve ended up getting nicked… Something to send a message that it was a false alarm would be good… otherwise we would be wasting a lot of ambulances’ times, like we would be up and gone by the time they get there probably, and there could be someone else that might really need it* [ambulance]*…like maybe it could be cancelled if our heartbeat returned to normal again for at least one minute… it could maybe send a direct message, ‘crisis averted’”* (Service User 12, male).

#### Appearance of wearable device

All respondents agreed the wearable device should be as discreet as possible and should resemble a plain wristband rather than a watch with an interface. The purpose and function of the device should not be obvious to others as they felt most users did not want relatives/friends to know they were using Heroin. The consensus was that it should appear as a thin black strap. It was also noted the device would need to be waterproof as a proportion of the target population would often be sleeping outdoors in all weathers.

#### Sense of safety

It was discussed whether the participants felt that wearing the device would provide them with a sense of safety (in that it would reduce the risk of dying from an overdose) and thus result in them taking more Heroin. The general view was that they did not use more Heroin when carrying Naloxone on their person and so did not perceive the wearing of the device would result in an increase in use.

*“Nah, you’re gonna use what you’re gonna use anyway as you’ve got Naloxone, users carry Naloxone, ya don’t use more if you’ve got that so why would ya use more with one of them on your wrist?”* (Service User 14, male).

#### Polydrug use

Two participants questioned whether the device would be able to detect a potential overdose when under the influence of any drug or was it just specifically designed to reduce the risk of Heroin overdose. All participants stated they often used other substances alongside Heroin, the main ones being Cocaine, Pregabalin (Lyrica) and/or Diazepam 10 mg (known locally as Blues).

*“Most of us who use Heroin ye know, like coke and blues goes side by side with it and tablets…ya know, those three things, we all do them… like we’re all proper junkies”* (Service User 5, female).

Over half (10 out of 16) considered the mixture of Pregabalin and/or Diazepam along with Heroin to be the trigger for an overdose. One respondent described the consequences of her using all three drugs simultaneously:

*“I took blues, Pregabalin and Heroin together and I collapsed… and the blood circulation stopped runnin’ through my hand and now it doesn’t work properly… I can’t move some of my fingers, they’re stuck like that…”* (Service User 4, female).

Facilitators explained the device was designed to detect a drop in the blood Oxygen saturation levels and heart rate which would be the indicator of an overdose from any substance or mixture of drugs and this would therefore still be of benefit to polydrug users.

#### Benefits vs. risks

A risk identified by some was that Naloxone could be administered to a person when not actually in an Opioid overdose situation. Others (*n* = 3) described their own experiences of having overdosed and why and ultimately they felt that the benefits of the device outweighed the risks:

*“I’ll tell you why this is a brilliant idea, cause if everyone panics and runs off and is willing to leave ya, at least the device will alert someone that you’re in trouble…my friend, who overdosed there in the public toilets, if she had one of them on her, someone could’ve tended to her but now she’s dead…”* (Service User 11, female).

The fact the users could not always depend on their peers to help them in an overdose situation was the main reason why they considered the device to be particularly beneficial. Education and information regarding Naloxone and the fact it can be used to help people in an overdose and significantly reduce the need for the police intervention was deemed vital:

*The main reason why people leave is because they don’t want the police to come, so when people finally understand that by administering Naloxone, the police aren’t gonna come because they’re bringing the person back… then they’re gonna use it… the only reason they run is because they’ve got warrants or because they don’t want lifted* [arrested] *or whatever… that’s why you’re better off to have one them on your arm….so as you have a better chance of getting help”* (Service User 3, female).

The wearable device was considered by all respondents to be most advantageous to those who tend to “shoot up” (inject drugs) alone at home or elsewhere:

*“If you’re on your own and you overdose somewhere in a flat or outside somewhere and nobody knows where ya are, like, then it would be good so as someone could find ya…I think it would be great for someone on their own….”* (Service User 2, female).

### Focus group with workers from street injector support service

A focus group was also conducted (remotely) with staff (*n* = 4) from Extern Street Injectors Support Service (SISS) to ascertain the views of workers regarding the feasibility of the wearables device and to obtain an overview of what the protocol is in a general overdose situation. Overall, the staff considered the device to be a good idea in principle, which would be beneficial in terms of harm reduction as it would help to reduce the number of deaths within the target population. They did, however, have some concerns regarding the practicalities of the design and the use of a wearable device within the drug using community. These are outlined in detail below.

#### Resale value

It was reiterated by staff that the device should not have a resale value, otherwise it would be sold or stolen. The workers noted that due to the chaotic lifestyle of some users, they would also have concerns as to whether they would lose the device and would have to be issued with a new wearable on a regular basis.

#### GPS tracking

All workers envisaged concerns and fears from users that authorities could potentially tap into the GPS system on the device, track their whereabouts on a daily basis and see the locations where they go to meet their dealers and buy drugs. They also anticipated that the dealers might have concerns and paranoia regarding their clients wearing the technology, which in turn, they feared could put the users at risk. All workers agreed that rumors can circulate quickly within the drug using community and if one person were to say that the device could track the user on a regular basis, this speculation would likely spread quickly throughout the drug using community and dealers. It was suggested by the SISS workers that the provision of honest and open information as to how the device would function was vital and could be relayed to the service users via their workers and other relevant frontline services.

#### Linkage with Northern Ireland ambulance service

From the workers experience, service users have had the chance to build up trust and rapport with the SISS team and on previous overdose occasions they did not respond well to the arrival of paramedics. One respondent reported that in the majority of times, when they had administered Naloxone in an overdose situation, and paramedics had arrived, the user had not allowed the paramedics to treat them as they felt that it was an intrusion.

*“They really do take offence… you know, when we bring them round with Naloxone, the majority of them will not let the paramedics work on them because they just don’t want that intrusion”* (Worker 1, female).

All SISS participants agreed that it would still be beneficial to alert the ambulance service as ultimately it was the decision of the user as to whether or not they wished to be taken to hospital in the ambulance.

*“The main thing is getting an alert to a possible overdose and getting Naloxone into them and it’s their decision ultimately as to whether they want to go to hospital or not, if they don’t want to, and they’re ok, well then their lives will still have technically been saved by the device and the Naloxone and yes it might wear off but there are other services in town, including ourselves that can monitor that”* (Worker 2, female).

#### Detection of specific substance

One suggestion was that the device could be modified to detect what type of substance the user had taken to cause the overdose, for example, benzodiazepines or heroin. They felt that this would be of great advantage as they also witness a significant number of overdoses due to Benzodiazepines, Street Benzos, Cocaine and Pregabalin.

#### Alert multiple sources

It was agreed by all workers that it would be beneficial to send an alert from the device to SISS and perhaps to a nominated friend of the user. This would ensure that the user would receive a quick response from whoever is within the closest proximity. The workers pointed out that as they are based in the city center and travel on bicycles, that their response time would likely be quicker than the ambulance service. They would also be able to administer Naloxone ahead of the arrival of the emergency services.

#### Ambivalence of drug users

All staff expressed concern that some users may remove the device when injecting as they would not want to potentially ruin their “hit” with the administration of Naloxone. Workers stated that a significant number of their service users were not concerned whether they lived or died, and the “hit” was paramount, regardless of the consequences.

*“…the majority of them doesn’t want you* [sic] *to inject them with Naloxone, so it’s like that mind set where they’ll think, ‘well I’ve a device on me, that’s maybe going to send a thing saying I need an injection of Naloxone, I’m gonna take it off”* (Worker 1, female).

## Findings 2: wearable phase

For the feasibility wearable phase of the research, the Withings ScanWatch was chosen as the data collection device after a thorough survey of the consumer wearable products/services available in the current markets including Apple, Samsung, Fitbit, Garmin and Huawei. Huawei band 2, although provides 24/7 SPo2 and heart rate measurement, does not provide data export out of the ecosystem. Most devices from other brands did not provide full control of the SPo2 measurement (SPo2 measurement with other devices is triggered only during the night, while a user is sleeping). Therefore, the Withings Brand was the only product that could meet the following requirements:

1. Collect both heart rate and Oxygen saturation (SPo2) readings from Opioid users with full control of the data.
2. Collect both heart rate and SPo2 data and synchronized to a cloud service in real-time.
3. Both heart rate and SPo2 data would be stored and could easily be accessible to use for later analysis.

During the feasibility study, participants were required to wear a ScanWatch under a shared Withings Health 10 mate account. Although not having their separate accounts created, each user’s data was tagged and could be identified by his/her ScanWatch serial ID. In this way, all users’ data was managed by a single account, hence making the subsequent data sharing process much easier and controllable.

**Figure 1:**
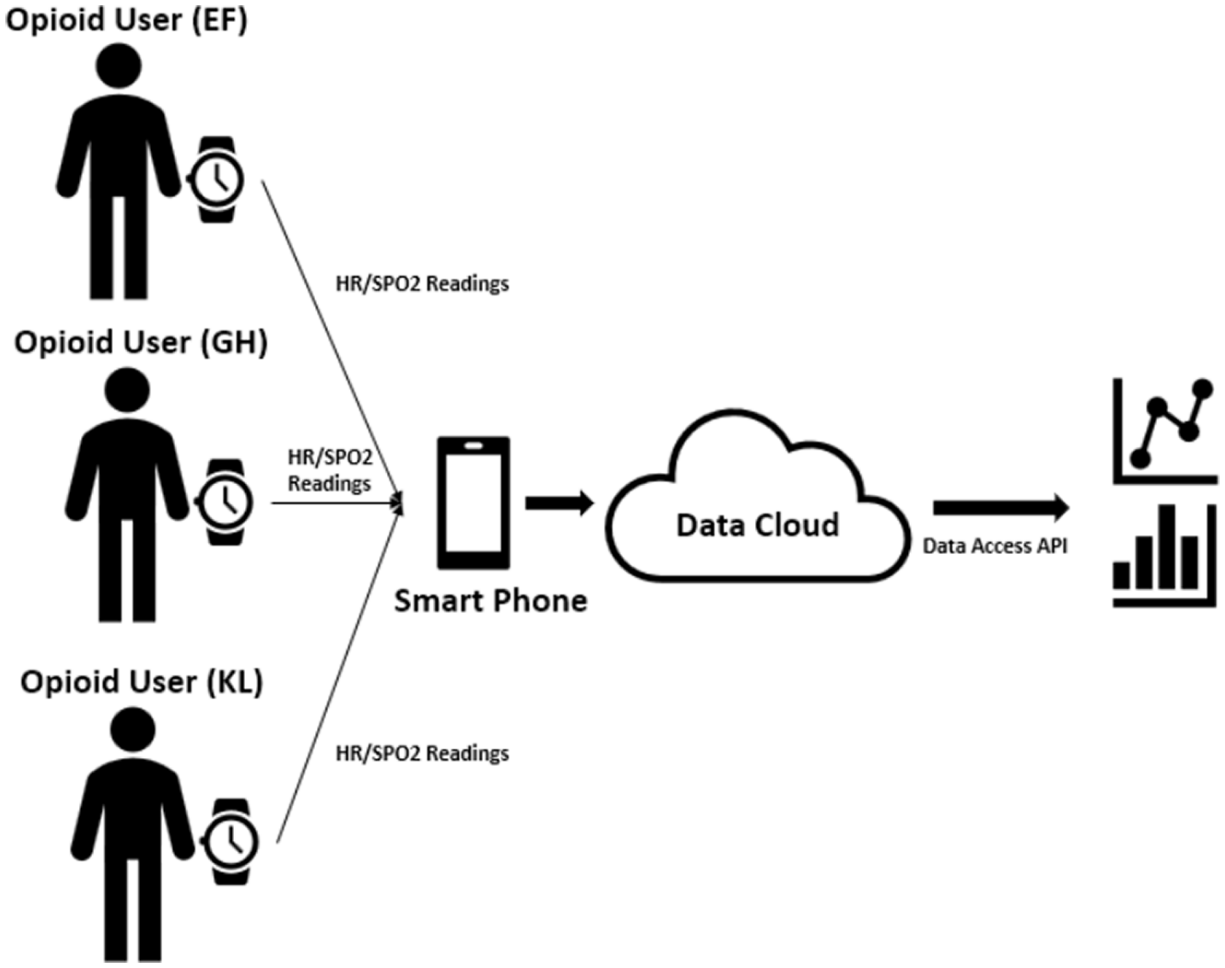
The data collection process and the system architecture.

The ScanWatch regularly and automatically (each minute) measured the users’ heart rate without requiring any user inputs. While for the Oxygen saturation readings, users did need to manually trigger the SPo2 measuring mode and hold the watch for 30 s to make sure a valid SPo2 reading is generated. This was not considered a major drawback at this stage as the aim was to test feasibility of the transferability of data to a secure server. Thus, the need to manually trigger the SPo2 reading was not an issue as the device was not being used for real-time detection of an overdose situation. Both measured data were then synchronized through Bluetooth from the watch to a smartphone device that acted as a hub and transferred all the watch data to the backend cloud service. Through the data access API provided by the data cloud service, our analyst was able retrieve the information and perform local data exploration and analysis (see Figure 1).

After all the data was retrieved for the feasibility study, an initial data exploration study was conducted with a local analysis tool kit (Python). From this exploration, we are confident to conclude that it is feasible to use a consumable wearable device for monitoring Opioid users’ biomarkers remotely. The data acquisition and transfer process will not be a barrier for such studies going forward.

## Discussion

### Focus group findings

The purpose of the qualitative component from the co-production phase was to provide information on the views and experiences of Opioid users regarding the acceptability and feasibility of wearing the device to preserve life in a potential overdose situation and to capture any ideas of how best to improve the design. In doing so, this research contributed to theoretical approaches which guide the use of digital technologies that enable intensive longitudinal data collection, tailoring to participants’ specific needs, or delivering targeted feedback or prompts in response to changing conditions (22, 23).

The vast majority of participants (from PWID groups and the worker group) favored the benefits of a wearable device, which significantly outweighed the risks. They seemed keen to avail of the technology as soon as it was fully developed. It was agreed that information about the wearable device and how it worked needed to be readily accessible and distributed clearly to users via frontline services. This was considered key, particularly in relation to the GPS function. It was clear from the findings that users were keen to know exactly how the device would work before they would wear it. Education regarding how the device would trigger the alert was therefore crucial in order to secure buy-in and trust from the target population.

Other ideas emerging from the focus groups with PWID related to the size and flexibility of strap, the appearance of the device and the importance of having an alarm to alert passers-by as well as being linked in with a first responder service. When asked about how to best distribute the device to the target population, the consensus was that distribution should take place from a community-based facility. Users suggested the device could be readily available within a city center facility such as SISS and that they could receive a device when collecting needle packs and Naloxone. In light of this, this article adds to a growing body of literature that shows how the potential for digital interventions, such as wearable technologies, can help avoid opiate overdose (2, 16–19). Overall, SISS workers agreed that the device would be of great benefit in principle to users, particularly in terms of harm reduction. They did, however, share their views and ideas regarding the further refinement of the device for future development. Key points raised by staff included the importance of education and availability of information regarding the functioning of the device, the importance of alerting others as well as the ambulance service to ensure best response time and administration of Naloxone. SISS workers felt that their service could play a pivotal role in the initiative, as regards the dissemination of information about the technology and distribution of the device itself within the drug using community. It would also be most efficacious in responding to the alert of a potential overdose situation in the quickest time in order to administer Naloxone.

GPS tracking was the greatest concern shared in all focus groups and by both PWID and workers. In the context of Covid-19 this would be a salient area for future research. As this research was conducted in the early stages of the Covid-19 pandemic questions of tracking relating to Covid-19 were left unexplored. Initial findings around GPS-tracking from this study demonstrated concerns regarding safety about being tracked in criminalised behaviors (where participants buy drugs/where they inject/if the device is noticed by dealers). As “track and trace” became a fundamental, heavily criticised (26), part of Covid-related public health policy in the UK (27), there is an opportunity for future research to examine how PWID feel about any possible implications of being GPS-tracked in a lockdown setting (when moving around is prohibited, or moving to or from certain areas is prohibited). Further research could potentially shape PWID interest and willingness to use the device at a time when the information about their movements or location can be very sensitive.

### Wearable phase data

The heart rate and Oxygen saturations are basic metrics and can be used in conjunction with other parameters to determine the acuity of illness and as a trigger for an intervention such as the administration of Naloxone. It was therefore considered essential that the wearable device that was used in this feasibility study could capture both blood Oxygen saturation levels and heart rate. It was concluded that the blood Oxygen saturation levels and heart rate measured by the ScanWatch device had been successfully recorded and transferred to the secure cloud service which could easily be accessed by the research team. This suggests the possible use of wearable devices to provide a safety profile for patients who use drugs and is in keeping with a successful proof of concept.

As this study sought to assess whether the data (SPo2 and heart rate) recorded on the device could be successfully transferred from the device to a backend cloud server and was not testing the device in a real time overdose situation, the manual recording of SPo2 readings using the Withings ScanWatch was not a significant issue. Despite this, the researchers noted a number of shortfalls in using the Withings ScanWatch as a data collection device in general. The SPo2 reading was difficult to capture, and the readings were often inconclusive. If there was even a slight movement from the participant, the reading was deemed to be inconclusive. In addition, if the users’ hands and wrists were cold, the device failed to capture Oxygen saturation levels as this resulted in a low pulsatile signal. It is quite common for users who inject to have circulatory issues which may, however, explain this issue. There were also a significant number of occasions during data collection in which the SPo2 readings were below average (between 83 and 93%). Normal pulse oximeter readings usually range from 95 to 100%.

It was noted throughout all service user focus groups that the Withings ScanWatch device was unsuitable, as it required the user to place their hand over the screen in order to obtain a reading of SPo2 in a potential overdose situation. It was agreed the user would be unable to perform this action in any potential life-threatening situation and it was unanimously agreed by participants that it would be necessary for both the readings for heart rate and SPo2 to be monitored automatically by the device without any intervention from the user.

## Conclusion

The study captured the views of Opioid users regarding the acceptability and practicality of wearing a device for PWID to detect the signs of an overdose. Those who took part offered important insight into the potential benefits and risks associated with wearing the technology within the drug using community as well as a vision of what the device should look like and how it should function in order for it to succeed. On the basis of these findings, future research could focus on the development, refinement, and piloting of a wearable device fit for purpose within the drug using community. Such findings could help address further crises if innovative digital solutions such as wearable technology are used to prevent Opioid-related deaths. Emergencies often present unexpected and uninvited ways of hastening solutions to problems, which we have wrestled with for decades. In the current post-Covid phase, we are not fully aware of how the virus may change and mutate. Currently, a number of countries are experiencing an ongoing surge of the Omicron variant. However, there is the ongoing risk of future variants of concern, which may precipitate sequential lockdowns in specific regions across the globe. As we debate the post-Covid phase and as we move into a potential endemic phase over the next 2 years (28), we are mindful that people who use drugs are still at risk of the social isolation and loneliness associated with heroin overdose and the increased barriers to seeking emergency medical care. PWID are also always at risk of further isolation in the event of the emergence of more aggressive and transmissible Covid strains. Wearable technologies for overdose prevention may help to address some of the lasting impacts of the Covid-19 crisis (29) including the increasing drug related deaths caused by overdose in the UK and on an international basis.

### Limitations

The sample size was small and not representative of the target population in general. This was partly due to the Covid-19 pandemic, subsequent restrictions and also the complexity of recruiting those from within the prison and homeless population.

## Data availability statement

The original contributions presented in the study are included in the article/Supplementary material, further inquiries can be directed to the corresponding author.

## Ethics statement

The studies involving human participants were reviewed and approved by Queens University Belfast Ethics Committee. The patients/participants provided their written informed consent to participate in this study.

## Author contributions

AC: project administration, formal analysis, writing—original draft, and visualization. SM: formal analysis, investigation, and writing—review and editing. LG: data curation, formal analysis, and writing—review and editing. UJ: formal analysis and writing—review and editing. CR: writing—review and editing. AD: investigation and writing—review and editing. All authors contributed to the article and approved the submitted version.

## Funding

This study has been funded by the Economic and Social Research Council (ESRC) Impact Acceleration Account. The ESRC impact fund was adminstered by Queens University Belfast.

## Conflict of interest

The authors declare that the research was conducted in the absence of any commercial or financial relationships that could be construed as a potential conflict of interest.

## Publisher’s note

All claims expressed in this article are solely those of the authors and do not necessarily represent those of their affiliated organizations, or those of the publisher, the editors and the reviewers. Any product that may be evaluated in this article, or claim that may be made by its manufacturer, is not guaranteed or endorsed by the publisher.
